# Experiences of Augmentative and Alternative Communication use in School: A Qualitative Evidence Synthesis Protocol

**DOI:** 10.12688/hrbopenres.14332.2

**Published:** 2026-08-04

**Authors:** Ciara Byrnes, Geraldine Moran, Michael T. Clarke, Jessica Caron, Emma M. Smith, Rose Galvin, Aoife Lily Gallagher

**Affiliations:** 1 School of Allied Health, University of Limerick Faculty of Education and Health Sciences, Limerick, County Limerick, Ireland; 2 Health Research Institute, University of Limerick, Limerick, V94 T9PX, Ireland; 3 Communication Sciences and Disorders - CSD, The Pennsylvania State University College of Health and Human Development, University Park, Pennsylvania, 16802, USA

**Keywords:** Qualitative evidence synthesis; augmentative and alternative communication; communication devices; children; disability; AAC user; communication needs; school; inclusive education; implementation science.

## Abstract

**Background:**

Augmentative and Alternative Communication (AAC) refers to a range of communication methods used by children or adults to enhance or replace oral communication. Studies show many children with communication needs who require AAC do not have access to appropriate supports, and that embedding AAC interventions in the classroom is challenging. This means many children who use AAC face barriers to meaningful participation, engagement, and independence. Understanding the experiences and perspectives of AAC users and those who support the use of AAC in the classroom can provide actionable knowledge to guide service improvement such that AAC users can be empowered to achieve and participate in school.

**Methods:**

A comprehensive literature search of the following electronic databases will be completed: PubMed, CINAHL Complete, EMBASE, EBSCO and Scopus. A manual search of the grey literature will also be carried out. Qualitative studies and mixed-methods designs that include qualitative data on the perspectives and experiences of AAC users, their families, and educators and healthcare professionals who support AAC users in school settings for children 3–18 years will be analysed. Methodological quality of included papers will be appraised using the Critical Appraisal Skills Programme (CASP) checklist for qualitative research by two independent reviewers. The Grading of Recommendations Assessment, Development, and Evaluation (GRADE) - Confidence in the Evidence from Reviews of Qualitative research (CERQual) approach will be employed to assess how much confidence to place in the findings of the qualitative evidence synthesis. Qualitative content analysis will be undertaken using constructs from Normalisation Process Theory.

**Conclusion:**

This qualitative evidence synthesis will summarise findings from the empirical international literature to provide a deeper understanding of the experiences of AAC use from a range of stakeholder perspectives. Findings will inform the development of pathways to support implementation of AAC for children with communication needs within schools.

**Prospero Registration No.** CRD420251229480.

## Introduction

Children and young people with complex communication needs benefit from using augmentative and alternative communication (AAC) methods to enhance their communication and participation. Such methods can help overcome difficulties associated with absent, unreliable or unintelligible speech. ^1^ AAC can refer to a range of communication methods that are used to supplement or replace speech, these methods can be aided or unaided. ^2, 3^ Aided systems can include picture, communication books and boards, and computerised speech-output devices; unaided AAC does not require any equipment, and the person can use their body, e.g., through signing or gesturing, to communicate. AAC is a recognised means of communication under the United Nations Convention on the Rights of Persons with Disabilities (UNCRPD). Article 21 outlines the rights of persons with disabilities to be provided with, and supported in utilising, the most accessible form of communication for them, including AAC. ^4^

Previous studies highlight that many children do not receive timely access to AAC support, resulting in reduced opportunities to develop language, interact and participate in their daily lives. ^5^ A complex range of interacting factors at an individual and at a systems level are proposed to contribute to this gap in AAC services and supports. From the perspective of the AAC user, difficulties are reported in relation to the useability of devices, a high cognitive load when learning how to use a communication system, and poor physical and sensory access to devices and/or systems. ^6– 9^ Parents have described their experience in terms of a lack of emotional readiness and of feeling overwhelmed by implementation of the use of AAC with their child in the home. Some parents go on to describe the use of AAC as an additional burden of care. Unrealistic expectations and frustration with regards to the ongoing efforts required to implement AAC in the home can result in device abandonment. ^10^

At the systems level, a lack of clinical expertise on the part of speech and language therapists and teachers working with AAC users is reported. ^11, 12^ Other factors include a lack of congruency between the AAC user’s communication needs and the AAC system they are provided with, and difficulty accessing funding for more expensive systems. Reduced social networks and communication partners unfamiliar with AAC further compound AAC use for the child. ^5^ At a societal level, further attitudinal barriers are discussed in the literature, namely *speechism* defined as “prejudice and discrimination based on how, as well as whether, someone uses speech to express themselves”. ^13^

Dumitru (2025) noted a gap in the literature on the perceptions and viewpoints of teachers relating to AAC systems. ^14^ A small number of studies have started to explore the use of AAC use in the classroom from an implementation science perspective. Implementation science can bridge the gap between research and clinical practice for healthcare practitioners by examining the differences that exist between evidence-based practice and the reality of what is delivered clinically in practice. ^15^ Using an implementation science approach enables the researcher to give due consideration to the complex and multi-system factors that directly influence the uptake and commitment to evidence-based interventions for people in real time, leading to improved outcomes for service users. ^15, 16^ Research on AAC implementation frequently cites the burden of care on families, ^10^ use of the implementation science framework allows for consideration of the multiple interrelated ecological systems, as described by Bronfenbrenner ^17^ in which a child’s development exists. ^18^ By using a systematic approach to identify factors that influence and support AAC implementation in school settings, implementation science can drive practice change, which increases healthcare value both financially and in terms of patient and system outcomes. ^16^ Normalisation Process Theory (NPT) ^19^ can be applied to understand what other contextual factors contribute to the barriers and facilitators of AAC implementation and how these interplay with one another. Given that NPT is deemed useful for research involving people with long-term conditions, ^20^ it is appropriate in the analysis and interpretation of research relating to child AAC users, by aiding the understanding and implementation of complex interventions. ^21^

The primary aim of this qualitative evidence synthesis is to summarise findings from the empirical international literature with regards to the experiences and perspectives of AAC users, their families, educators and healthcare professionals about AAC use in the classroom. Given our lack of understanding and the complexity of implementing AAC interventions in the classroom, it is crucial to increase our knowledge and appreciation of the contextual factors in order to optimise successful outcomes for all involved.

The study will address the following research questions:

(i) How do stakeholders describe their experiences of the implementation of AAC in the classroom?
(ii) What factors influence implementation of AAC in the classroom setting?
(iii) What implementation strategies are required to support the use of AAC in the classroom setting?

## Method

### Study design

A qualitative evidence synthesis (QES) will be undertaken. A QES methodology involves a comprehensive systematic search of the research literature to identify, review and collate findings from existing qualitative research, and analyse this for the purpose of gaining an understanding of stakeholder experiences of the use of AAC for children with communication needs in the classroom. QES findings may support the development of guidelines in healthcare that are informed by the lived experiences of stakeholders. ^22^ A protocol for this QES is registered on PROSPERO (International prospective register of systematic reviews): CRD420251229480.

### Public patient involvement

A PPI panel has been established to support this qualitative evidence synthesis across all stages of the review. The relevance, quality and impact of this research will be enhanced by including the unique knowledge and experience of AAC users, their parents, educators and healthcare staff from the outset of this review. ^23^ The PPI group consists of a primary school principal, teacher, special needs assistant, speech and language therapist, parent and person with lived experience; all the individuals are familiar with AAC. The researcher will adhere to the guidance provided for including the unique voice of the child in the PPI group alongside the other representatives. ^24^ The PPI group have been engaged to review and refine the research question and inclusion and exclusion criteria. The group have also advised on issues relating to AAC implementation that are of importance to the wider community. The group will support with the data analysis and interpretation, by reviewing the outcomes of the synthesis to evaluate its relevance and reliability. Dissemination of findings will also be guided by the members of the PPI group, along with planning next steps.

### Search strategy

Guided by the SPIDER (Sample, Phenomenon of Interest, Design, Evaluation, and Research type) tool, ^25^ the search string for the databases was developed in conjunction with the University of Limerick’s Health Research Methods Librarian and is available as Table 1 *.* As inclusive education became a core principle for child education provision regardless of differences following the publication of the Salamanca Statement, ^26^ the literature search will be restricted from June 1994 to present day.

**Table 1.** Search strategy for pubmed.

S1	“communication devices for people with disabilities”[MeSH Terms] OR (“augmentative”[Title/Abstract] AND “alternative communication systems”[Title/Abstract]) OR “AAC”[Title/Abstract] OR “communication device”[Title/Abstract] OR “communication aid”[Title/Abstract] OR “speech generat* device”[Title/Abstract]
S2	“child”[MeSH Terms] OR “child, preschool”[MeSH Terms] OR “adolescent”[MeSH Terms] OR “child*”[Title/Abstract] OR “child preschool”[Title/Abstract] OR “adolescen*”[Title/Abstract]
S3	“educational status”[MeSH Terms] OR “education”[MeSH Terms] OR “school”[Title/Abstract] OR “educat*”[Title/Abstract] OR “primary”[Title/Abstract] OR “secondary*”[Title/Abstract] OR “elementary”[Title/Abstract] OR “class*”[Title/Abstract] OR “special education”[Title/Abstract] OR “class*”[Title/Abstract] OR “college”[Title/Abstract] OR “preschool”[Title/Abstract] OR “early years educat*”[Title/Abstract]
S4	S1 + S2 + S3

Key concepts and terms relevant to this QES are incorporated into the search strategy, namely augmentative and alternative communication, communication devices for people with disabilities, AAC, child, education, school, in addition to MeSH terms.

The following electronic sources databases will be searched: Pubmed, CINAHL Complete, EMBASE, EBSCO, and Scopus, will be accessed to identify relevant studies. A manual search of the grey literature will also be completed guided by the expertise of the research team and snowballing techniques. The following websites will be searched: SpeechBITE, ASHAWire, Communication Matters, The Informed SLP, Advances in Communication and Swallowing (Irish Association of Speech and Language Therapists, IASLT) and The International Journal of Language and Communication Disorders (Royal College of Speech and Language Therapist, RCSLT). The reference lists of included studies will also be searched to identify additional studies. When conducting grey literature searches, a record will be kept including the date of search, search terms and number of papers yielded. These will be added to the screening process and reported using the PRISMA guidelines. This will enhance replicability of grey literature searches.

**Study selection**

The inclusion criteria were defined using the SPIDER acronym. ^25^ Studies will be considered for inclusion if they report on:

**S**ample: School children aged between 3–18 years who use AAC; parents and/or caregivers, education and healthcare professionals with experience supporting school children who use AAC.

**P**henomenon of **I**nterest: All studies reporting on stakeholder experiences of AAC use in school.

**D**esign: All published and unpublished qualitative and mixed-methods studies including theses, whereby the qualitative data can be extracted.

**E**valuation of outcomes: Themes derived from the qualitative or narrative data that are representative of the sample’s perspectives and experiences of AAC use and implementation for school-going children with CCN between 3–18 years.

**R**esearch Type: Peer reviewed journal articles and theses, from 01 June 1994 to present will be included. There will be no restrictions based on the language of the publication.

**Augmentative and Alternative Communication (AAC),** defined as a broad range of methods which support a person with communication needs or differences to communicate with others, particularly when speech is unreliable. We will include studies that report on both unaided or aided AAC. Unaided AAC involves the use of the person’s body to communicate, including using gestures, eye gaze, and sign language whereas aided AAC require tools and/or equipment such as photographs, pictures and communication boards, switches and speech-generating devices. We will include studies where AAC is used to enhance or replace a person’s natural speech and provide opportunities to develop language, communication and interaction skills. ^27^

**Population:** The population of interest includes children and young people from 3–18 years in school settings who use AAC for communication, their family members, caregivers as well as school staff (school principals, teachers and teaching assistants/special needs assistants) and healthcare staff (speech and language therapists) who provide AAC support.

**Study types:** Primary qualitative investigations (e.g., grounded theory, phenomenology, qualitative descriptive studies) employing established methods of qualitative data collection (such as interviews) and recognised approaches to qualitative data synthesis will be eligible for inclusion. Survey designs incorporating free-text response options may also be considered, provided that the qualitative data generated demonstrates sufficient depth and has been subjected to formal analysis using a structured methodology (e.g., thematic analysis, content analysis). In the case of mixed-method studies, only the qualitative component in which participants’ experiences are reported separately will be included. Opinion pieces and commentaries will not be considered for inclusion due to the risk of bias and lack of transparent, replicable methodology.

### Screening process

All studies from the initial searches will be imported into Covidence, a reference management system, where duplicates will be removed. Two authors (CB and GM) will independently screen the titles and abstracts of all studies in Covidence to identify studies suitable for inclusion. A third author (AG) will be consulted to screen the titles and abstracts if consensus cannot be reached.

The full texts of articles will be retrieved where they are deemed eligible for inclusion or where there is insufficient information in the abstract to permit a decision on the article’s relevance. These full text articles will be reviewed by two authors (CB and GM) in relation to their relevance for inclusion in the QES. A third author will be consulted where a decision on inclusion cannot be reached. Levels of agreement (%) across these phases of screening will be reported. A record will be retained of reasons for exclusion of papers at full text review and reported in the final QES in a PRISMA flow diagram. ^28^

### Quality appraisal of the included studies

The methodological quality of the included studies will be appraised using the 10-item Critical Appraisal Skills Programme ^29^ checklist for qualitative studies (CASP, 2022). The CASP is a concise and effective tool for critical appraisal of evidence and supports the reader to consider three key elements when reviewing qualitative studies: firstly, are the results of the review valid, secondly what are the results, and finally, will the results help locally. ^30, 31^ Three authors (CB, RG and AG) will independently assess the quality of each study against this checklist. Disagreements will be managed through discussion or involvement of a further member of the research team (GM). No studies will be excluded based on quality; the appraisal will be used to identify methodological shortcomings in the conduct and/or interpretation of the study findings. Significant methodological weaknesses will be contextualised throughout the discussion of the paper.

**Assessing the confidence of the QES findings**

The Grading of Recommendations Assessment, Development, and Evaluation (GRADE) - Confidence in the Evidence from Reviews of Qualitative research (CERQual) approach will be employed by the researcher to evaluate and summarise the quality of the studies. ^32^ The findings will be contextualised in relation to the quality appraisal of the included of studies.

### Data extraction and synthesis

CB will extract data from each individual study using a custom pre-piloted data extraction form created with Covidence. This will include information on the authors, year of publication, country where the study was conducted, stakeholder group(s) studied, characteristics of the AAC systems, the educational context, data collection setting, sampling method, approach to data collection, and key findings. These findings will be narratively described and presented in a table of descriptive characteristics.

The included papers will be imported into NVivo Version 15 (NVivo 2024) for analysis. NVivo is a tool that facilitates the research team to store, organise and code qualitative data using a systematic approach, as well as to record analytical decisions. ^33^ Inductive content analysis will be used to identify first level open codes across the dataset within the included literature. ^34^ The second level coding involves application of the four domains of Normalisation Process Theory (NPT). The four domains of NPT – coherence, cognitive participation, collective action, and reflexive monitoring – will be used deductively to understand how AAC practices have or have not become routine practice in the classroom. ^21^ These four domains support us in establishing how a new intervention in understood by the people using it (coherence), how committed and engaged people are with the intervention (cognitive participation), what people have to do to provide or engage with an intervention (collective action), and finally, how people evaluate or appraise the impact of an intervention (reflexive monitoring). ^21^

**Reflexivity**

Given the significance of a researcher’s own stance on their research, a robust qualitative design requires the researcher to reflect on their influence or subjective bias and document how this may shape their findings. ^35, 36^ To ensure rigour and transparency, a research diary will be used by the researcher to document, reflect and acknowledge changes in approaches and understanding as a result of the research process. The reflexivity process will be iterative, and journalling will occur alongside regular peer-supervision with the research team to record discussions and reflections.

### Dissemination of findings

On completion of the analysis, it is anticipated that the report of the completed study will be submitted for publication in a peer reviewed journal. Findings will also be disseminated to AAC users, their families, educators and the speech and language therapists, national policy makers and organisations who represent the interests of AAC users. The PPI panel will co-write a lay summary of the findings to support dissemination of the findings and will be invited to collaborate with the researcher in sharing the findings upon completion.

### Study status

Incomplete.

## Discussion

This review will synthesise the existing evidence, as well as add to the empirical literature in relation to stakeholders’ experiences and perceptions of AAC implementation for children with communication needs in schools.

## Ethics and consent

Ethical approval and consent were not required.

## Data Availability

No data are associated with this article. A completed PRISMA-P checklist ^37^ is available at https://doi.org/10.17605/OSF.IO/PQDA7. This work is licensed under Creative Commons Attribution 4.0 International. To view a copy of this license, visit https://creativecommons.org/licenses/by/4.0/. ^38^ Open Science Framework: PRISMA-P Checklist for ‘Experiences of Augmentative and Alternative Communication use in School: Findings from a Qualitative Evidence Synthesis’: The conduct and reporting of this QES will adhere to the ENTREQ guidelines (Enhancing Transparency in Reporting the Synthesis of Qualitative Research), ^39^ the 21-item checklist designed to enhance the quality and reporting of qualitative evidence syntheses. A copy of this checklist will be included with the completed QES.
