# Supplementary material for: Experiences of Augmentative and Alternative Communication use in School: A Qualitative Evidence Synthesis Protocol
Source: HRB Open Res. 2026 Aug 4;9:42. Originally published 2026 Apr 28. [Version 2] doi: 10.12688/hrbopenres.14332.2 (PMC13550906; doi:10.12688/hrbopenres.14332.2)
Supplement: Peer review history

# Reviewer report: Reviewer response for version 2

**DOI:** 10.21956/hrbopenres.16003.r57062

**Published:** 2026-08-07

**Authors:** Cristina Dumitru

**Affiliations:** 1 The National University of Science and Technology POLITEHNICA Bucharest, Pitești University Centre, Pitesti, Romania

The article was improved significantly, I do not have any other comments.

Is the study design appropriate for the research question?

Yes

Is the rationale for, and objectives of, the study clearly described?

Yes

Are sufficient details of the methods provided to allow replication by others?

Partly

Are the datasets clearly presented in a useable and accessible format?

Not applicable

Reviewer Expertise:

Education, Inclusive education, Neuroeducation

I confirm that I have read this submission and believe that I have an appropriate level of expertise to confirm that it is of an acceptable scientific standard.

# Reviewer report: Reviewer response for version 1

**DOI:** 10.21956/hrbopenres.15783.r55911

**Published:** 2026-07-22

**Authors:** Sylwia Kazmierczak-Murray, Laura Gormley

**Affiliations:** 1 Dublin City University Institute of Education, Dublin, Leinster, Ireland

**Peer review by: **Dr Sylwia Kazmierczak-Murray & Dr Laura Gormley, School of Inclusive and Special Education, Institute of Education, Dublin City University, Ireland 

 This is a timely protocol that addresses an important and underexplored area of research. Understanding the implementation of augmentative and alternative communication (AAC) in educational settings is highly relevant to improving participation and educational outcomes for children who use AAC. The protocol demonstrates several strengths, including thoughtful membership of the public patient involvement (PPI) panel, soundness of the methodological process, and the application of an implementation science framework to guide the interpretation of the findings. The proposed evidence synthesis will make a very valuable contribution to the field. There are a few areas where additional clarification and/or methodological justification could be considered. We outline these areas below. 

**Justification for the use of Normalisation Process Theory (NPT)**

 The selection of Normalisation Process Theory (NPT) is novel and potentially valuable; however, the rationale for its use could be strengthened. NPT focuses on understanding the social processes through which practices become embedded, enacted and sustained in routine settings. However, the review questions include identifying factors influencing implementation and determining what implementation strategies are required to support AAC use in classrooms. NPT does not directly function as a determinant framework, nor does it explicitly identify implementation strategies. Clarification regarding the relationship between the review objectives and the explanatory mechanisms offered by NPT would be a helpful addition.

 Implementation research in schools consistently highlights the importance of school-level factors, including leadership, collaborative capacity, professional learning opportunities, time allocation and resource availability, which may be unique to individual schools and are often perceived differently by the stakeholders not based in schools. In this context, while the inclusion of AAC users, family members, educators and healthcare professionals is valuable, further consideration should be given to how collective constructs within NPT will be applied across stakeholder groups with potentially divergent experiences and priorities, given that the review is focused on ‘normalisation’ of AAC within educational contexts, not society more broadly. The authors could consider whether NPT concepts such as coherence and cognitive participation can be meaningfully interpreted across teachers, parents, healthcare professionals and AAC users collectively, or whether stakeholder-specific analyses may be required.

**Clarification of the proposed qualitative synthesis approach**

 The decision to begin the search from 1994 is justified by reference to the Salamanca Statement and developments in inclusive education. However, this rationale may not fully align with the realities of how communication differences are understood and supported across contexts. Many children who use AAC are educated in special rather than inclusive educational environments. The authors could consider whether a later date, such as the international adoption of the United Nations Convention on the Rights of Persons with Disabilities (UNCRPD) in 2006, would provide a more relevant context.

 Thoughtful consideration has already been given to the search protocol and it is positive to see a focus on the grey literature. This aspect would be further enhanced by providing more detailed information on the search terms to be used and how these terms will be combined, for the database searches. Furthermore, clarification of how the grey literature will be searched (e.g., sources to be searched, terms to be used) would be beneficial, as well as what it means, operationally, when the authors state that this aspect will be guided by the expertise of the research team.

 Greater clarity regarding the approach to double screening and consensus procedures could be reported, given not just the interpretive nature of this research, but also the specialist expertise required to identify relevant AAC studies. Reference and adherence to the Enhancing Transparency in Reporting the Synthesis of Qualitative Research (ENTREQ) guidelines (Tong et al., 2012) might be helpful in this respect. Furthermore, consideration should be given to conducting a reliability check for the data extraction process for at least one-third of the included articles.

 The data analysis section would benefit from further elaboration. The authors state that inductive content analysis will be used initially to identify and define codes within the included studies, followed by deductive mapping to the four NPT domains: coherence, cognitive participation, collective action and reflexive monitoring. The precise analytical steps between the inductive and deductive stages would benefit from further explanation (e.g., how findings that do not align with NPT constructs will be handled).

**Considerations for the interpretation of findings**

 The protocol would benefit from consideration of how the specificity of educational context across countries (e.g., between Global North versus Global South) will be incorporated into the synthesis and interpretation of findings. Attitudes toward disability and communication differences, inclusive education policy, access to AAC, availability of specialist support, and teacher professional learning vary substantially across countries. These differences have significant implications for implementation processes and the barriers or facilitators identified. The authors should clarify whether contextual factors will be extracted and considered during synthesis and how they will address challenges related to transferability across highly diverse educational systems.

 The protocol does not indicate whether distinctions between AAC modalities will be made, especially between powered and non-powered AAC systems. Emerging evidence suggests that the barriers, facilitators and implementation supports associated with these AAC modalities may differ in schools. Clarification regarding whether this distinction will be considered during data extraction or synthesis would be helpful to include.

 While the scope of the review is commendable, the authors should clarify how the differential experiences of the different stakeholders (e.g., AAC users, educational professionals, parents, healthcare professionals) will be managed and addressed in the data extraction and synthesis process, as well as the interpretation of findings.

**Terminology considerations**

 As previously mentioned, the inclusion of a PPI is a strength of this protocol. To further enhance this aspect and given the fact that the review has an educational focus, authors should consider adapting the terminology to represent an educational context, as opposed to a more medicalised context. For example, authors could use terms such as ‘Research Advisory Panel’ or ‘Research Advisory Group’.

Is the study design appropriate for the research question?

Yes

Is the rationale for, and objectives of, the study clearly described?

Yes

Are sufficient details of the methods provided to allow replication by others?

Yes

Are the datasets clearly presented in a useable and accessible format?

Not applicable

Reviewer Expertise:

Inclusive and Special Education

We confirm that we have read this submission and believe that we have an appropriate level of expertise to confirm that it is of an acceptable scientific standard.

## References

[1] : Enhancing transparency in reporting the synthesis of qualitative research: ENTREQ. *BMC Medical Research Methodology* .2012;12(1) : 10.1186/1471-2288-12-181 10.1186/1471-2288-12-181 23185978 PMC3552766

# Reviewer report: Reviewer response for version 1

**DOI:** 10.21956/hrbopenres.15783.r55906

**Published:** 2026-07-06

**Authors:** Jorge Eduardo Alfaro-Urrutia

**Affiliations:** 1 Universidad San Sebastián, Puerto Montt, Chile

The protocol addresses a relevant and timely topic, as understanding how Augmentative and Alternative Communication (AAC) is incorporated into everyday classroom practices constitutes a challenge for inclusive education.

 The research question is clearly formulated, and the protocol presents a solid methodological design, with procedures that promote transparency and rigor in the review. However, it would be advisable to clarify how the proposed deductive analysis will be carried out. Although the elements to be extracted from the studies are described (for example, authors, year of publication, context, participants, and main findings), the a priori analytical categories that will guide the synthesis are not specified. Considering that the objectives of the review focus on the experiences of the factors involved, the factors that influence the implementation of AAC, and the strategies necessary to promote its use in the classroom, it would be advisable to define or explain (not necessarily in a theoretical manner) more precisely which dimensions or constructs are expected to be identified within each of these domains.

 For example, within experiences, it would be useful to clarify whether these include training experiences, experiences implementing AAC, experiences observing implementations, or all of these. This would contribute to strengthening the transparency, reproducibility, and coherence of the proposed deductive approach. Likewise, this would help guide the data extraction process. Congratulations on the work you are carrying out; I am sure it will be a valuable contribution.

Is the study design appropriate for the research question?

Yes

Is the rationale for, and objectives of, the study clearly described?

Yes

Are sufficient details of the methods provided to allow replication by others?

Yes

Are the datasets clearly presented in a useable and accessible format?

Yes

Reviewer Expertise:

AAC in autism, families and teachers

I confirm that I have read this submission and believe that I have an appropriate level of expertise to confirm that it is of an acceptable scientific standard.

# Response

**Published:** 2026-07-14

**Authors:** Ciara Byrnes

**Affiliations:** School of Allied Health, University of Limerick Faculty of Education and Health Sciences, Limerick, County Limerick, Ireland

Thank you so much for your time for considering our draft protocol. We hope that we have addressed your point comprehensively below.

 We have amended the data extraction process to include data on implementation strategies which goes someways towards addressing this point. This will allow us to contextualise the experiences that we will synthesise inductively across the data set during the first level of coding.

# Reviewer report: Reviewer response for version 1

**DOI:** 10.21956/hrbopenres.15783.r55907

**Published:** 2026-06-29

**Authors:** Cristina Dumitru

**Affiliations:** 1 The National University of Science and Technology POLITEHNICA Bucharest, Pitești University Centre, Pitesti, Romania

Dear Editor,

 Thank you for the opportunity to review this protocol entitled "Experiences and perspectives of augmentative and alternative communication (AAC) use in educational settings: a qualitative evidence synthesis protocol."

 This protocol addresses an important gap in the literature by proposing a qualitative evidence synthesis exploring the experiences of AAC users, families, educators, and healthcare professionals regarding AAC implementation in educational settings. The study is use of Normalisation Process Theory (NPT), PROSPERO registration, SPIDER search strategy, CASP quality appraisal, GRADE-CERQual, and public and patient involvement (PPI) demonstrates methodological rigor and transparency.

 Overall, I believe the protocol has the potential to make a valuable contribution to implementation science and inclusive education research. Nevertheless, several issues should be addressed before indexation. 

 1. Explain why NPT was chosen instead of the Consolidated Framework for Implementation Research (CFIR), RE-AIM, or the Theoretical Domains Framework (TDF).

 2. The review is restricted to English-language publications. Acknowledge this as a limitation because important qualitative studies on AAC may have been published in other languages.

 3.Although several grey literature sources are listed, the search strategy could be described in greater detail. Specify search procedures, inclusion criteria, methods used to identify unpublished studies and how grey literature quality will be evaluated.

 My recommendation is Minor Revision.

 4. Some terminology could be standardized throughout the manuscript. For example: use either "AAC users" or "children who use AAC" consistently; "education settings" or "school settings" consistently.

 The protocol states that no studies will be excluded based on CASP quality assessment. Clarify how studies with major methodological limitations will influence the interpretation of findings during CERQual assessment.

 5. The planned extraction table is appropriate. Consider also extracting characteristics of AAC systems, educational context, implementation strategies, reported barriers, reported facilitators, theoretical frameworks used in the original studies.

 The authors may wish to consider including the recent systematic review by Dumitru (2025), which synthesizes current evidence on the implementation of assistive technology and augmentative and alternative communication (AAC) in educational settings. This review provides an up-to-date overview of barriers, facilitators, and the effects of AAC on language development, making it highly relevant to the background and discussion of the proposed qualitative evidence synthesis. 

 Sincerely,

Is the study design appropriate for the research question?

Yes

Is the rationale for, and objectives of, the study clearly described?

Yes

Are sufficient details of the methods provided to allow replication by others?

Partly

Are the datasets clearly presented in a useable and accessible format?

Not applicable

Reviewer Expertise:

Education, Inclusive education, Neuroeducation

I confirm that I have read this submission and believe that I have an appropriate level of expertise to confirm that it is of an acceptable scientific standard.

## References

[1] : Assistive Technology and Alternative and Augmentative Communication Options in the Language Skills Development of Students with Specific Learning Disorders. *Education Sciences* .2025;15(2) : 10.3390/educsci15020170 10.3390/educsci15020170

# Response

**Published:** 2026-07-14

**Authors:** Ciara Byrnes

**Affiliations:** School of Allied Health, University of Limerick Faculty of Education and Health Sciences, Limerick, County Limerick, Ireland

Thank you so much for your time for considering this protocol. We have made amendments to reflect your very valuable feedback, see below:

 Regarding your first point, we acknowledge that there are several theories and frameworks that could have been applied; NPT was chosen as a comprehensive theory with a specific focus on collective social action and collaboration, which we feel better supports research that examines implementing change within a school and classroom. We know that interprofessional collaboration has been explored in the literature and proven to be very challenging, therefore the collective action element of NPT is important in this context across health and education.

 For point two, we have removed the English language criteria; thank you for raising this important issue with the protocol. This will ensure important publications in other languages and not overlooked.

 Regarding point three, we have expanded the detail regarding grey literature searches as follows: when conducting grey literature searches a record will be kept including date of search, search terms and number of papers yielded. These will be added to the screening process and reported using the PRISMA guidelines. This will enhance replicability of grey literature searches.

 Point four regarding the standardisation of terminology is now actioned throughout the manuscript. Thank you for bringing this to our attention.

 In relation to point five regarding extraction: thank you for these helpful comments, we will add all bar barriers and facilitators, as we are conducting inductive coding of the papers informed by implementation science theory which will provide us with a more nuanced understanding of implementation factors.

 Finally, thank you for sharing your study with us, we did not include this paper in the literature background as it is primarily outcome focused, however we have referenced your study in justifying the gap in the literature in the introduction.
